# *Sinistroporomonorchis bolini* n. sp. (Trematoda: Monorchiidae) from the Ocellated killifish, *Floridichthys polyommus* (Cyprinodontidae) in the Yucatán Peninsula, Mexico based on an integrative taxonomy analysis

**DOI:** 10.1017/S0031182025000010

**Published:** 2025-01

**Authors:** Leopoldo Andrade-Gómez, Juan F. Espínola-Novelo, Brenda Solórzano-García, Gerardo Pérez-Ponce de León

**Affiliations:** Departamento de Sistemas y Procesos Naturales, Escuela Nacional de Estudios Superiores Unidad Mérida, Yucatán, Mexico

**Keywords:** 28S, *cox*1, digenea, helminth, PCA, phylogeny, SEM

## Abstract

Members of *Sinistroporomonorchis* Wee, Cutmore, Pérez-del-Olmo & Cribb, 2020 represent a small group of trematodes belonging to the Monorchiidae Odhner, 1911 with 5 species described from mugilid hosts. Specimens consistent with the generic concept of *Sinistroporomonorchis* were obtained from *Floridichthys polyommus* (Cyprinodontidae); most of them were juveniles from 4 localities within the Yucatán Peninsula. After a detailed morphological examination including scanning electron microscopy images and a principal component analysis, the specimens collected represented a new species, *Sinistroporomonorchis bolini* n. sp. The new species can be differentiated by the presence of an overall large pharynx including the proportion of pharynx width to oral sucker width, a uterus arranged in 2 main lateral fields, and by presenting robust caeca. In addition, sequences of the 28S of large subunit of nuclear ribosomal RNA and *cox*1 of the mitochondrial DNA were obtained. Phylogenetic trees inferred from each dataset, placed all the specimens in a monophyletic clade, confirming that the isolates belonged to the same species. The new species is the sixth described for the genus *Sinistroporomonorchis*, the fifth described from the Yucatán Peninsula and the first described from a non-mugilid host.

## Graphical abstract

**Figure:**
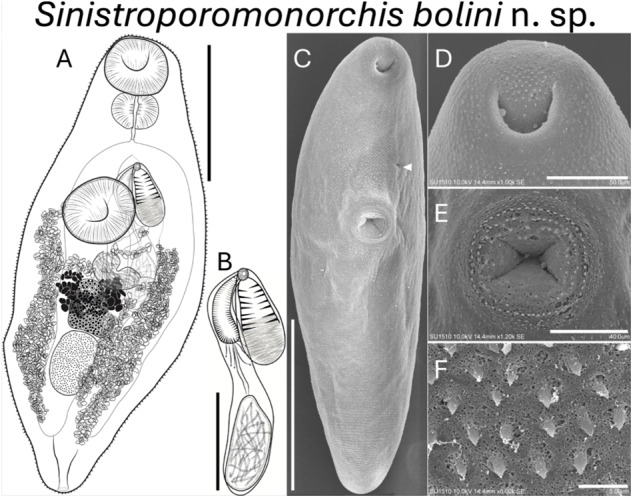

## Introduction

The genus *Sinistroporomonorchis* Wee, Cutmore, Pérez-del-Olmo & Cribb, 2020 is a small group of trematodes belonging to the Monorchiidae Odhner, 1911 with 5 species described thus far (Andrade-Gómez et al., 2023). This genus was proposed by Wee et al. (2020a) to include 2 species, *Sinistroporomonorchis glebulentus* (Overstreet, 1971) and *S. lizae* (Liu, 2002); both species are parasites of mugilids. The distinguishing characters that separate the genus from most other monorchiids is the possession of a distinctly sinistral genital pore, an unspecialized oral sucker, short oesophagus and restricted vitelline masses (Wee et al., 2020a). More recently, Andrade-Gómez et al. (2023) performed morphological and molecular analyses including the 28S region of the rDNA and *cox*1 of the mitochondrial DNA to describe 3 species of *Sinistroporomonorchis* from mugilids in the Yucatán Peninsula, Mexico, namely *S. mexicanus* (Andrade-Gómez et al., 2023); *S. yucatanensis* (Andrade-Gómez et al., 2023) and *S. minutus* (Andrade-Gómez et al., 2023).

Four of the five species were described from *Mugil* spp. (Mugilidae) in the Americas whereas *S. lizae* was described from *Planiliza carinata* (Valenciennes) (Mugilidae) in Asia (Overstreet, 1971; Liu, 2002; Andrade-Gómez et al., 2023). However, Andrade-Gómez et al. (2023) reported that *Lasiotocus* sp. ex *Menidia menidia* (*L.*) (Atherinopsidae) from New Jersey, USA sequenced by Panyi et al. (2020), should be considered as a member of *Sinistroporomonorchis* based on results of the 28S analysis (see Andrade-Gómez et al., 2023). Additionally, Espínola-Novelo et al. (2023, 2025) reported *Sinistroporomonorchis* sp. based on the sequence data of 28S rDNA of one individual obtained from *Floridichthys polyommus* (Hubbs) and one from *Fundulus persimilis* (Miller) in La Carbonera coastal lagoon, Yucatán, Mexico.

In the present study, we are completing the inventory of the parasite fauna of the *F. polyommus*; newly collected individuals of trematodes consistent with the concept of *Sinistroporomonorchis* were obtained from *F. polyommus* in 3 coastal lagoons and 1 locality off the coast of the Yucatán Peninsula. A detailed morphological examination includinga principal component analysis (PCA) of those specimens as well as scanning electron microscopy (SEM) photomicrographs, in combination with information from nuclear and mitochondrial DNA suggested that these specimens represented a new species of *Sinistroporomonorchis*. The new species is described herein.

## Materials and methods

### Sample collection

**Figure 1.**
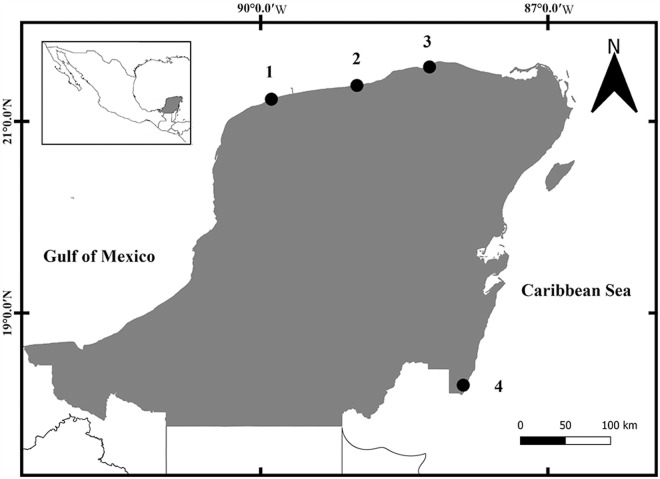
Sampling localities within the coastal lagoons of the Yucatán Peninsula, Mexico. Localities correspond with Table 1.

A total of 119 individuals from *F. polyommus* and 41 individuals from *F. persimilis* were collected using cast nets from 3 coastal lagoons and 1 locality off the coast the Yucatán Peninsula (YP) between 2023 and 2024 (Table 1; Figure 1). Fishes were kept alive and examined for helminths a few hours after capture. Individual fish was euthanized following the procedures accepted by the American Veterinary Medical Association (AVMA, 2020), dissected and immediately examined under a stereomicroscope. Sixty-two individuals of *Sinistroporomonorchis* were recovered from the intestines of 28 specimens of *F. polyommus*, and only 1 individual was recovered from an individual of *F. persimilis* (Espínola-Novelo et al., 2025) (Table 1). Most of the sampled specimens of *Sinistroporomonorchis* were juveniles (56%). Monorchiids were fixed in hot distilled water and preserved in 100% ethanol for morphological and molecular analysis.

**Table 1.** Locality, collection date, host species, host length, prevalence, intensity of infection and GenBank accession numbers of specimens collected in this study

	Locality	Georeference	Collection date	Host	TL (cm) ± s.d.	HI/HR	IoI	28S	*cox*1
1	La Carbonera, Yucatán	21°13ʹ54ʺN; 89°53ʹ25ʺW	May 2023	*Floridichthys polyommus*	6.5 ± 1.15	4/40	1	PQ634759	PQ630635
			September 2023	*Fundulus persimilis*	10.2 ± 2.9	1/20	1	PP919655*	–
2	Dzilam de Bravo, Yucatán	21°22ʹ31ʺN; 88°59ʹ44ʺW	June 2024	*Floridichthys polyommus*	9.2 ± 0.8	19/21	1–11	PQ634760	PQ630636
			October 2023	*Fundulus persimilis*	8.6 ± 1.8	0/21	–	–	–
3	San Felipe, Yucatán	21°34ʹ06ʺN 88°14ʹ13ʺW	January 2024	*Floridichthys polyommus*	6.8 ± 0.9	4/24	1–9	PQ634761–763	–
4	Off the coast of Xcalak, Quintana Roo	18°14ʹ32ʺN 87°53ʹ06ʺW	November 2023	*Floridichthys polyommus*	5.1 ± 0.8	1/34	1	PQ634764	PQ630637

TL, mean total length of hosts; HI/HR, host infected/host revised; IoI, intensity of infection.

Numbers for localities correspond to Figure 1.

* Published in Espínola-Novelo et al. (2025).

### Morphological analyses

Some specimens were post-fixed in hot formalin to harden the tegument. Posteriorly, those specimens were dehydrated through a graded alcohol series, stained with Mayer’s paracarmine (Merck, Darmstadt, Germany), cleared with methyl salicylate and mounted on microscope slides with Canada balsam. Mounted specimens were photographed, examined morphologically and measurements were obtained using under a bright field Nikon DS-Ri1 microscope with Nikon NIS Elements microscope software (Nikon). Measurements are given in micrometres (μm). Drawings were made with Adobe Illustrator 25.4.1 (Adobe, Inc.). Holotype and paratypes were deposited in the Colección Nacional de Helmintos (CNHE), Instituto de Biología, Universidad Nacional Autónoma de México, Mexico City.

For SEM, specimens were dehydrated in a graded ethanol series, critical point dried and sputter coated with gold. SEM images from the monorchiids were obtained with a Hitachi Stereoscan Model SU1510 SEM at 15 kV at the Laboratorio de Microscopia y Fotografía de la Biodiversidad, Instituto de Biología, Universidad Nacional Autónoma de México.

A PCA was implemented to explore and describe the patterns of morphological variation of 7 specimens of *Sinistroporomonorchis* collected from *F. polyommus*. Morphometrical data of the 23 specimens reported by Andrade-Gómez et al. (2023) belonging to 4 species, *Sinistroporomonorchis glebulentus, S. mexicanus, S. minutus* and *S. yucatanensis* (CNHE 11838–11844) were used to complement this analysis (Supplementary Table S1). The species *S. lizae* was not included in the analysis since we have no access to the original measurements, necessary to run the analysis. The PCA was conducted using ggplot2, ggfortify, cluster and lfda implemented in R (R Core Team, 2020) and included 58 metrical data (see Supplementary Table S1).

### Amplification and sequencing of DNA

Six specimens of *Sinistroporomonorchis* sp. collected from *F. polyommus* were placed individually in tubes with a digestion solution for DNA extraction at 56°C overnight. The digestion procedure, amplification and sequencing protocols followed Andrade-Gómez et al. (2023). The domains D1–D3 of the large subunit of nuclear ribosomal RNA gene (28S) were amplified *via* PCR using the primers: 391 5ʹ-AGCGGAGGAAAAGAAACTAA-3ʹ and 536: 5ʹ-CAGCTATCCTGAGG GAAAC-3ʹ for 28S (García-Varela and Nadler, 2005). Additionally, a fragment of *cox*1 was amplified for 2 specimens. In addition, the individual of *Sinistroporomonorchis* sp. sequenced by Espínola-Novelo et al. (2023) for 28S (OR537912) was sequenced for *cox*1 in the present study. For amplifying the *cox*1 region, the primers designed by McNamara et al. (2014) were used, i.e. cox1tremF (5ʹ-TTCACKTTGGATCATAAGCGT-3ʹ) and Mon.mt3 (5ʹ-ACCATAAACATRTGRTG-3ʹ). Sequences were assembled and edited using Geneious v7 (Kearse et al., 2012) and deposited in the GenBank database.

### Alignments and phylogenetic analyses

Sequences obtained in the current research from 28S rDNA and *cox*1 mtDNA were aligned separately with data from 5 species of *Sinistroporomonorchis* plus 3 unidentified sequences of *Sinistroporomonorchis*, 1 from *Menidia menidia* (MN984477), 1 from *F. polyommus* (OR537912) and 1 from *F. persimilis* (PP919655), all downloaded from the GenBank dataset. Three species of *Allobacciger* Hafeezullah & Siddiqi, 1970, one of *Alloinfundiburictus* Wee, Cutmore, Pérez-del-Olmo & Cribb, 2020 and one of *Monorchis* Monticelli, 1893 were used as outgroups (see Table 2). The alignments were trimmed to the shortest. The 28S rDNA alignment consisted of 46 sequences with 1186 nucleotides and 19 sequences with 496 nucleotides for *cox*1. Alignments were constructed using the software Clustal W (Thompson et al., 1997) with default parameters and adjusted manually with the Mesquite software (Maddison and Maddison, 2011).

**Table 2.** Sequences from GenBank used for phylogenetic analysis in the present study

Species	Host	Locality	28S	*cox*1	Reference
*Allobacciger annulatus* Wee, Cutmore, Sasal & Cribb, 2020	*Centropyge tibicen* (Cuvier)	Australia	MK955782	MK975248	Wee et al. (2020b)
*Allobacciger brevicirrus* Wee, Cutmore, Sasal & Cribb, 2020	*Scolopsis bilineata* (Bloch)	Australia	MK955781	MK975247	Wee et al. (2020b)
*Allobacciger polynesiensis* Wee, Cutmore, Sasal & Cribb, 2020	*Centropyge flavissima* (Cuvier)	French Polynesia	MK955780	JN969510	Wee et al. (2020b)
*Alloinfundiburictus haemuli* (Overstreet, 1969)	*Haemulon plumierii* (Lacèpede)	Mexico: Gulf of Mexico	OQ672280–83	OQ674197–98	Andrade-Gómez et al. (2023)
*Monorchis monorchis* (Stossich, 1890)	*Diplodus vulgaris* (Saint-Hilaire)	Mediterranean Sea	AF184257		Tkach et al. (2001)
*Sinistroporomonorchis* sp.	*Menidia menidia* (L.)	USA: Great Bay Estuary	MN984477		Panyi et al. (2020)
*Sinistroporomonorchis* sp.	*Floridichthys polyommus* (Hubbs)	Mexico: Yucatan, Gulf of Mexico	OR537912		Espínola-Novelo et al. (2023)
*Sinistroporomonorchis* sp.	*Fundulus persimilis* (Miller)	Mexico: Yucatan, Gulf of Mexico	PP919655		Espínola-Novelo et al. (2025)
*Sinistroporomonorchis glebulentus* (Overstreet, 1971)	*Mugil curema* (Valenciennes)	USA: Beufort; Mexico: Gulf of Mexico	MN984476; OQ672284–88	OQ674199–201	Panyi et al. (2020); Andrade-Gómez et al. (2023)
*Sinistroporomonorchis lizae* (Liu, 2002)	*Liza longimanus* (Günther)	Vietnam: Tonkin Bay	LN831720		Atopkin et al. (2017)
*Sinistroporomonorchis mexicanus* (Andrade-Gómez et al., 2023)	*Mugil curema*	Mexico: Gulf of Mexico	OQ672289–95	OQ674202–04	Andrade-Gómez et al. (2023)
	*Mugil* sp.		OQ672296		
*Sinistroporomonorchis yucatanensis* (Andrade-Gómez et al., 2023)	*Mugil curema*	Mexico: Gulf of Mexico	OQ672297–304	OQ674205–07	Andrade-Gómez et al. (2023)
*Sinistroporomonorchis minutus* (Andrade-Gómez et al., 2023)	*Mugil curema*	Mexico: Gulf of Mexico	OQ672306–10	OQ674209	Andrade-Gómez et al. (2023)
	*Mugil* sp.		OQ672305	OQ674208	

The phylogenetic analyses were performed using Bayesian inference (BI) and maximum likelihood (ML) methods. The BI analysis was inferred with MrBayes version 3.2.7 (Huelsenbeck and Ronquist, 2012) through the interface: Cyberinfrastructure for Phylogenetic Research Science Gateway v3.3 (CIPRES) (Miller et al., 2010) and the ML analysis were carried out with the RAxML version 7.0.4 (Silvestro and Michalak, 2011). The best model was estimated with the Akaike information criterion using the jModelTest version 0.1.1 program (Posada, 2008). The best model for each dataset was GTR + I + G. The BI analyses included 2 simultaneous runs of Markov chain Monte Carlo for 10 million generations, sampling every 1000 generations, a heating parameter value of 0.2 and a ‘burn-in’ of 25%. To support each node in the ML analyses, 10 000 bootstrap replicates were run. Trees were drawn using FigTree program v.1.3.1 (Rambaut, 2012). The genetic divergence among taxa was estimated using uncorrected ‘p’ distances in MEGA v. 6. (Tamura et al., 2013) (see Table 3).

**Table 3.** Pairwise nucleotide sequence comparisons between taxa for the aligned 28S rDNA sequences (1186 nt) below the diagonal and for *cox*1 sequences (496 nt) above the diagonal

28S/*cox*1	1 *Sinistroporomonorchis* sp.	2 *S. lizae*	3 *S. glebulentus*	4 *S. mexicanus*	5 *S. minutus*	6 *S. yucatanensis*	7 *S. bolini* n. sp.
1 *Sinistroporomonorchis* sp.	–	–	–	–	–	–	–
2 *S. lizae*	5.2	–	–	–	–	–	–
3 *S. glebulentus*	4.5	2.1–2.2	**0/0.2–0.8**	9–9.5	15.9–16.4	14.9–15.6	17.7–19.5
4 *S. mexicanus*	4.8	2.7–2.8	**1**	**0/0**	16.5–17.2	14.9–15.1	19.3–20
5 *S. minutus*	5.5–5.7	6.4–6.5	5.8–6	6.1–6.4	**0–0.17/9.2**	14–15.5	18.3–22
6 *S. yucatanensis*	5.8–6	6.9–7.2	6.3–6.5	6.8–7	2.7–2.9	**0–0.1/0.2–1.2**	17.9–20.6
7 *S. bolini* n. sp.	4.1–5.6	4.7–7.1	4.6–6.7	4.6–6.3	6.4–8.5	6.6–9.1	**0–1.4/0.8–11.46**

Bold represented the genetic intraspecific divergence.

## Results

### Taxonomy

Platyhelminthes Minot, 1876

Trematoda Rudolphi, 1808

Plagiorchiida La Rue, 1957

Monorchiidae Odhner, 1911

*Sinistroporomonorchis* Wee, Cutmore, Pérez-del-Olmo & Cribb, 2020

**Figure 2.**
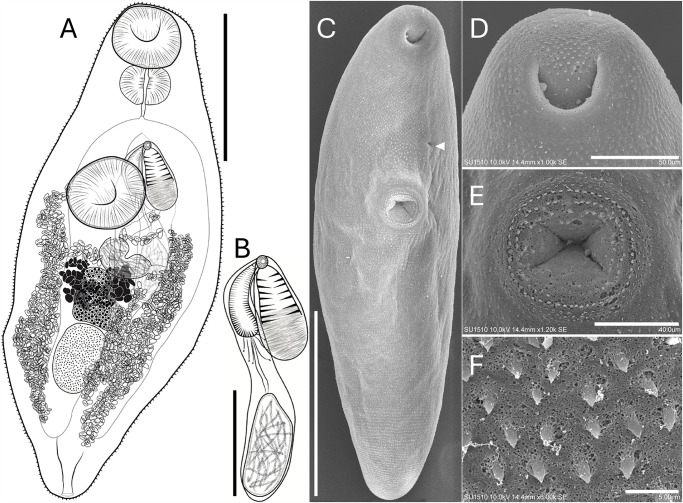
*Sinistroporomonorchis bolini* n. sp. from *Floridichthys polyommus*. (A) Whole worm holotype, ventral view; (B) terminal genitalia of holotype; scanning electron micrographs of voucher; (C) whole worm; (D) oral sucker; (E) ventral sucker; (F) tegumental spines. Scale bars (μm) = (A) 200; (B) 100; (C) 300; (D) 50; (E) 40; (F) 5. Arrow marks genital pore.

*Sinistroporomonorchis bolini* n. sp. (Figure 2)

**Type host**: *Floridichthys polyommus* Hubbs, Ocellated killifish, Cyprinodontidae

**Additional host**: *Fundulus persimilis* Miller, Yucatán Killifish, Fundulidae

**Site of infection**: Intestine

**Type locality**: Dzilam de Bravo, Yucatán (21°22ʹ31ʺN; 88°59ʹ44ʺW)

**Additional localities**: La Carbonera (21°13ʹ54ʺN; 89°53ʹ25ʺW) and San Felipe (21°34ʹ06ʺN; 88°14ʹ13ʺW) lagoons, Yucatán; Off the coast of Xcalak (18°14ʹ32ʺN; 87°53ʹ06ʺW), Quintana Roo.

**Specimens deposited**: 1 Holotype (CNHE-12163); 6 paratypes (CNHE-12164)

**Etymology**: The specific epithet *bolini* refers to the vernacular name of the host, *Floridichthys polyommus* which is locally known as ‘bolin yucateco’. We treat the word ‘bolin’ as a masculine proper name.

**GenBank Accession:** PQ634759*–*PQ634764 for 28S; PQ630635*–*PQ630637 for *cox*1

**ZooBank LSID**: 0A32C2A1-8B29-4D7C-A9C5-B57263159BDA

***Sinistroporomonorchis bolini* n. sp.** (Figure 2).

## Description

Based on 7 mature individuals (Range followed by the mean in parentheses). Body fusiform 561–742 (675) long, 175–288 (216) wide, widest at midbody; 2.4–3.7 (3.1) times longer than wide. Tegument thin, armed with small spines throughout body. Forebody relatively short 209–262 (226) long, occupies 29.6–37.6 (33.7)% of body length. Hindbody 282–424 (354) long, occupies 49.7–57.1 (52.4)% of body length. Oral sucker spherical, terminal, with ventral aperture, 56–91 (76) long, 57–101 (83) wide. Ventral sucker round located anterior to middle of body, 65–128 (91) long, 68–108 (83) wide. Oral sucker length to ventral sucker length ratio 1: 0.64–1.01 (0.84). Oral sucker width to ventral sucker width ratio 1: 0.83–1.13 (0.97). Prepharynx observed in some specimens, 2–8 (5) long. Pharynx well-developed, spherical, large, 41–59 (51) long, 57–84 (71) wide; 58.8–88.5 (68.3)% of oral sucker length; 75.2–100 (87.3)% of oral sucker width. Oesophagus short 26–90 (54) long, occupies 3.6–12.5 (7.9)% of body length. Intestinal bifurcation in forebody, well anterior to ventral sucker; pre-bifurcal zone 100–221 (162) long, occupies 13.4–30.7 (24)% of body length. Intestinal caeca robust, long, terminate posterior to testis. Post-testicular zone 34–161 (102) long, or 6–22.5 (14.9)% of body length from posterior end of body.

Testis single, ovoid, 80–126 (105) long, 52–81 (62) wide; posterior to midbody, 14.3–20.7 (17.9)% of body length from ventral sucker, 84–154 (121). Pre-testicular zone 365–480 (433) long, occupies 59.7–66.8 (64.2)% of body length; post-testicular zone 109–166 (136) long, or 16.8–23.8 (20.2)% of body length. Cirrus-sac arcuate, dorsal to ventral sucker extending to middle of body, mostly intercaecal, slightly overlaps left caecum, 169–243 (203) long, 27–45 (34) wide, occupies 26.1–34.3 (30.6)% of body length. Seminal vesicle elongate, unipartite, 25–101 (54) long, 22–46 (37) wide; occupies 12.4–41.5 (26.6)% of cirrus-sac length. Pars prostatica simple, with few prostatic cells observed, 24–37 (33) long. Cirrus thick, armed with spines, 74–145 (108) long, 15–32 (20) wide, occupies 40.3–77.1 (53.6)% of cirrus-sac length. Genital atrium unspined. Common genital pore small, sinistral to midline, anterior to ventral sucker, located close to intestinal bifurcation.

Ovary lobulate, slightly irregular and elongate transversally, in anterior half of hindbody, distinctly posterior to ventral sucker, overlaps right caecum ventrally, 40–89 (67) long, 45–78 (57) wide; pre-ovarian zone 145–421 (341) long, or 24.8–58.6 (50.8)% of body length; post-ovarian zone 138–289 (225) long, or 23.7–41.5 (33.7)% of body length. Vitellarium composed of 2 dense clusters of follicles that are confluent at the posterior half of the clusters, irregularly shaped, distributed closely and dorsal to ovary, 45–78 (63) long or 7.7–10.9 (9.3)% of body length. Uterus mostly restricted to hindbody in lateral fields of the body, thin-walled, extensive, ventral to ovary, testis, caeca and part of cirrus-sac, with coils mostly indiscernible; ascending coil forms metraterm and enters terminal organ at posterior end. Terminal organ sinistro-ventral to cirrus-sac, 86–120 (100) long, 41–78 (53) wide, comprising unspined posterior chamber, and spined anterior section. Posterior chamber spherical, containing fibrous mass 26–51 (36) long, 33–55 (44) wide. Anterior section armed with long and thin spines, 47–85 (63) long, 24–61 (61) wide. Seminal receptacle canalicular, anterior to ovary. Eggs slightly tanned, operculate, unfilamented, 7–19 (12) long, 3–9 (7) wide. Excretory vesicle Y-shaped. Excretory pore terminal.

## Morphometric analyses

**Figure 3.**
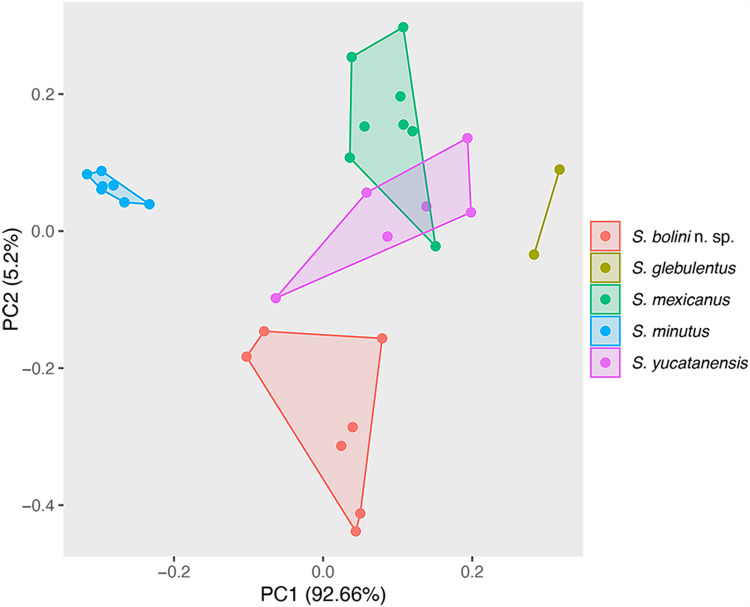
Principal component analysis of 5 species of *Sinistroporomonorchis* conducted with 58 morphometric variables from 30 individuals.

PCA was conducted to corroborate the morphological differences between the new species and those previously described by Andrade-Gómez et al. (2023). A total of 58 variables were considered from 30 specimens corresponding to 5 species, *S. bolini* n. sp., *S. glebulentus, S. minutus, S. mexicanus* and *S. yucatanensis.* The morphometrical data of the new species from *F. polyommus* formed a separate cluster from the other species, particularly along PC2, corroborating the separation of this species from the other congeners. In addition, *S. minutus* was also separated, as well as the 2 individuals of *S. glebulentus*. Only 1 individual from *S. mexicanus* was grouped with *S. yucatanensis* (Figure 3).

## Remarks

The new species, *S. bolini* n. sp. collected from *F. polyommus* in 4 localities of the Yucatán Peninsula, Mexico is consistent with the generic concept of *Sinistroporomonorchis* in the possession of a sinistral genital pore, an unspecialized oral sucker, and restricted vitelline masses. With the addition of the new species, the genus *Sinistroporomonorchis* now contains 6 species. The newly described species overlaps in most of the metrical characters in comparison with the remaining 5 species. However, *S. bolini* n. sp. can be differentiated from the 5 *Sinistroporomonorchis* species by the presence of an overall larger pharynx; in the new species pharynx is 41–59 (51) long and 57–84 (71) wide, whereas the range of the other 5 species varies between 22 and 46 long and 30–55 wide. Likewise, the proportion of pharynx width to oral sucker width could also be a reliable character to distinguish the new species, 0.75–1 (0.87); meanwhile, the range of the other 5 species is 0.41–0.84. Additionally, *S. bolini* n. sp. can be further differentiated from the other congeners by possessing a uterus arranged in 2 main lateral fields overlapping the caeca; in addition, caeca are very wide (robust); all other *Sinistroporomonorchis* spp. possess simple caeca and the uterus is not distributed in 2 lateral fields. Furthermore, in the PCA, the individuals of the new species formed a separated cluster from the other species of *Sinistroporomonorchis*. This result supports the validity of the new species.

## Molecular data and phylogenetic analysis

### 28S

**Figure 4.**
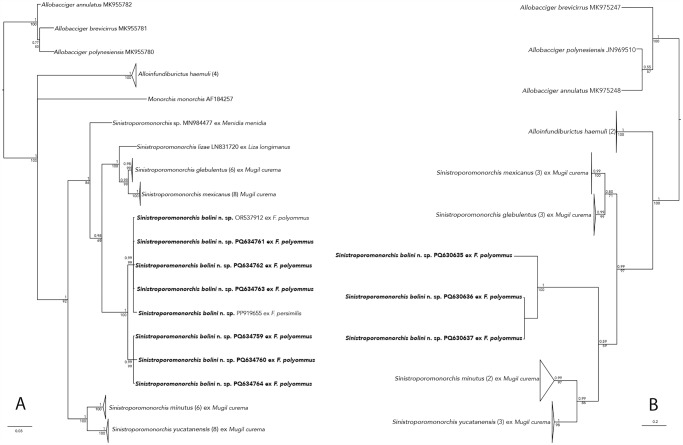
Phylogenetic trees inferred from the Bayesian inference and maximum likelihood analyses of the (A) 28S gene and (B) *cox*1 gene. Posterior probabilities (BI) and bootstrap values (ML) are shown above and below the nodes, respectively.

The phylogenetic analyses inferred with ML and BI recovered similar topologies (Figure 4A). The genus *Sinistroporomonorchis* was yielded as monophyletic, with high nodal support although it formed an unresolved clade with *Monorchis* and *Alloinfundiburictus* with strong support (1/100). Within the genus *Sinistroporomonorchis*, 2 subclades were formed. The first major subclade contained *Sinistroporomonorchis* sp. (MN984477), as the sister taxa of 2 groups, one containing *S. lizae*, and *S. glebulentus* + *S. mexicanus*, and the other containing the isolates of *S. bolini* n. sp. with high to moderate nodal support (1/86). The second major subclade was formed by *Sinistroporomonorchis minutus* + *S. yucatanensis,* with strong support (1/100). The 6 new sequences of *S. bolini* n. sp. from *F. polyommus*, plus the 2 sequences (OR537912, PP919655) obtained by Espínola-Novelo et al. (2023); Espínola-Novelo et al. (2025) were nested within a clade with strong support (1/100) corroborating the conspecifity, although some isolates grouped in 2 separate groups, but with very low sequenced divergence. The new species was recovered as the sister taxa of the clade formed by *S. lizae*, and *S. glebulentus* + *S. mexicanus* with strong to moderate nodal support (0.98/68).

The genetic divergence estimated for 28S rDNA between the new species and all other representative species of *Sinistroporomonorchis* ranged from 4.1 to 9.1%. The genetic divergence between the new species and the closely related species (*S. lizae, S. glebulentus* and *S. mexicanus*) ranged from 4.6 to 7.1%. The genetic divergence between the new species and the second subclade (*Sinistroporomonorchis minutus* + *S. yucatanensis*) is even higher, varying 6.4–9.1%. The intraspecific genetic divergence among the 8 new sequences of *S. bolini* n. sp. varied between 0 and 1.4% (Table 3).

### cox*1*

The phylogenetic analyses inferred with ML and BI also recovered similar topologies (Figure 4B), although all branches were resolved. The analyses also showed *Sinistroporomonorchis* resolved as monophyletic with high nodal support (0.99/97). Two subclades were also formed, however, unlike 28S analysis, the phylogenetic position of the new species was different. In the first subclade, *S. glebulentus* was yielded as the sister species of *S. mexicanus*. In the second subclade, the new species was nested as the sister taxa of *S. minutus* + *S. yucatanensis* albeit with very low support values (0.59/59). The 3 newly sequenced individuals of *S. bolini* n. sp. formed a separated clade with strong nodal support (1/100).

The genetic divergence estimated for *cox*1 between the new species and its sister taxa (*S. minutus* + *S. yucatanensis*) was 18.3–22%. The divergence between the new species and the other major subclade (*S. glebulentus* + *S. mexicanus*) was 17.7–20%. Finally, the intraspecific genetic variation among the 3 newly sequences of *S. bolini* n. sp. was 0.8–11.4% (Table 3).

## Discussion

Members of *Sinistroporomonorchis* are typically associated with mugilid hosts (Mugilidae). However, Andrade-Gómez et al. (2023) concluded that *Lasiotocus* sp. reported by Panyi et al. (2020) from the atherinopsid *Menidia menidia* should also be considered as a member of *Sinistroporomonorchis*. Espínola-Novelo et al. (2023) and Espínola-Novelo et al. (2025) then reported the presence of *Sinistroporomonorchis* sp. also from 2 non-mugilid species, the cyprinodontid *F. polyommus* and the fundulid *F. persimilis* in La Carbonera coastal lagoon, Yucatán Peninsula, Mexico but were unable to identify the species due to the limited number of specimens obtained from these hosts. This indicates that the genus can no longer be considered specific to mugilids, since apparently several host-switching events have occurred during the evolutionary history of the group.

In this study, following an integrative taxonomy approach, we describe *Sinistroporomonorchis bolini* n. sp. from *F. polyommus* and *F. persimilis* in 4 localities of the Yucatán Peninsula, Mexico. The new species is the sixth described for the genus *Sinistroporomonorchis* and the fifth described from the Yucatán Peninsula. It is worth noting that prevalence and abundance values of *S. bolini* n. sp. is relatively low compared with values of these ecological parameters obtained for other *Sinistroporomonorchis* species (see Overstreet, 1971; Liu, 2002; Andrade-Gómez et al., 2023); this may indicate that in fact mugilids are the main host group, and that the presence of species of *Sinistroporomonorchis* in other fish groups indicate a recent diversification. Also, only 1 individual of the new species was found in 41 sampled specimens of *F. persimilis* studied in 2 localities of the Yucatán Peninsula, suggesting this may be an accidental infection. Furthermore, most of the specimens of *S. bolini* n. sp. found in *F. polyommus* were juveniles which may indicate the novel development of the new species in a non-mugilid host that occur in sympatry with mugilids and is apparently experiencing an incipient diversification process. However, it is clear that the high genetic divergence observed between the new species and other congeners for both molecular markers confirmed the validity of the new species.

In this study, we recovered the same topology as in Andrade-Gómez et al. (2023) (Figure 4). However, the phylogenetic position of the new species with respect to the other congeners differed with each molecular marker. In the 28S analysis, *S. bolini* n. sp. was placed in the first subclade as sister group to a clade formed by *S. glebulentus, S. mexicanus* and *S. lizae*. Meanwhile, with *cox*1 analysis, *S. bolini* n. sp. was recovered in the second major subclade as the sister group of *S. minutus* and *S. yucatanensis*, although with weak nodal support. Based on these results, we consider that the 28S analysis provide, as in other trematodes, a better understanding of the evolutionary interrelationships among *Sinistroporomonorchis* as discussed by Pérez-Ponce de León and Hernández-Mena (2019).

Finally, this study represents an effort to continue the description of the diversity of parasites associated with *F. polyommus*. Still, more information is required to solve the phylogenetic position of the new species, and the evolutionary relationships of *Sinistroporomonorchis* species and most likely more species of this genus remain undiscovered since they might be associated to a variety of hosts, including species of atherinopsids, cyprinodontids and fundulids.

## Supporting information

Andrade-Gómez et al. supplementary material Andrade-Gómez et al. supplementary material

## Supplementary material

The supplementary material for this article can be found at https://doi.org/10.1017/S0031182025000010.
